# GPT2: Empirical slant delay model for radio space geodetic techniques

**DOI:** 10.1002/grl.50288

**Published:** 2013-03-22

**Authors:** K Lagler, M Schindelegger, J Böhm, H Krásná, T Nilsson

**Affiliations:** 1 Department of Geodesy and Geoinformation (Research Group Advanced Geodesy), Vienna University of TechnologyVienna, Austria; 2 Institute of Geodesy and Photogrammetry (Geosensors and Engineering Geodesy)ETH Zurich, Switzerland; 3 Section 1.1, GPS/GALILEO Earth Observation, Deutsches GeoForschungsZentrumPotsdam, Germany

## Abstract

Up to now, state-of-the-art empirical slant delay modeling for processing observations from radio space geodetic techniques has been provided by a combination of two empirical models. These are GPT (Global Pressure and Temperature) and GMF (Global Mapping Function), both operating on the basis of long-term averages of surface values from numerical weather models. Weaknesses in GPT/GMF, specifically their limited spatial and temporal variability, are largely eradicated by a new, combined model GPT2, which provides pressure, temperature, lapse rate, water vapor pressure, and mapping function coefficients at any site, resting upon a global 5° grid of mean values, annual, and semi-annual variations in all parameters. Built on ERA-Interim data, GPT2 brings forth improved empirical slant delays for geophysical studies. Compared to GPT/GMF, GPT2 yields a 40% reduction of annual and semi-annual amplitude differences in station heights with respect to a solution based on instantaneous local pressure values and the Vienna mapping functions 1, as shown with a series of global VLBI (Very Long Baseline Interferometry) solutions.

## 1. Introduction

Tropospheric slant delays used in the analysis of GNSS (Global Navigation Satellite System), VLBI (Very Long Baseline Interferometry), and DORIS (Doppler orbitography by radiopositioning integrated on satellite) observations are normally modeled as the sum of a hydrostatic and a wet part (Davis *et al*. 1985), each of them being the product of zenith delay and corresponding mapping function. Whereas wet zenith delays are usually estimated, hydrostatic zenith delays can be derived from the pressure value at the observation site following Saastamoinen (1972). Knowledge of the instantaneous local pressure arises from barometric recordings, the gridded surface pressure output of a numerical weather model (NWM), or global empirical models, which approximate the spatial and temporal pressure variability. Nowadays, the common empirical model used in GNSS/VLBI/DORIS processing is GPT (Global Pressure and Temperature) (Böhm *et al*. 2007); see Petit and Luzum (2010)).

Both hydrostatic and wet mapping functions are expressed by the coefficients {*a*,*b*,*c*} given in the continued fraction form of Herring (1992). These coefficients are different for the hydrostatic and wet mapping functions and can be calculated in several ways. Within the currently used models, like the Isobaric Mapping Function (IMF (Niell, 2001)) or the Vienna Mapping Function 1 (VMF1 (Böhm *et al*. 2006b)), the coefficients of the hydrostatic and wet terms are obtained from operational analysis and forecast fields of NWMs, and issued for download. If they are not accessible, one may deploy empirical mapping models that are based on average values derived from NWMs. The Global Mapping Function (GMF (Böhm *et al*. 2006a)), which depends only on the station coordinates and the day of year (doy), can be noted as such an auxiliary model.

There are some weaknesses to both models (GPT, GMF). These have been improved within a new combined model named GPT2. See Table 1 for a comprehensive overview. In the first place, GPT/GMF parameters are expanded to spherical harmonics of degree and order 9, leading to a coarse horizontal resolution of about 20°. Hence, the models’ capability of representing large height variations and the associated change of parameters is restricted. As a second issue, considerable height differences ( > 1 km) have to be dealt with when reducing meteorological quantities from the model surface to the actual station height. Within GPT2, a refined horizontal resolution of 5° partly compensates for these problems. The data used are monthly mean profiles of the latest ECMWF (European Centre for Medium-Range Weather Forecasts) Re-Analysis (ERA-Interim [*Dee et al.*, 2011]). Temporal coverage (2001–2010), vertical resolution (37 isobaric levels), and quality of the ERA-Interim data surpass the characteristics of ERA-40 fields [*Uppala et al.*, 2005] that were utilized for GPT/GMF (three years of monthly mean profiles at 23 isobaric levels). In those models, only mean and annual variation (phase fixed to 28 January) of the parameters were estimated within a least-squares adjustment at mean sea level. In addition to that, GPT2 incorporates semi-annual harmonics in order to better account for regions where very rainy periods or very dry periods dominate. Furthermore, GPT2 replaces GPT's constant temperature lapse rate of − 6.5°C/km by mean values and (semi-)annual variations of the temperature lapse rate at each grid point. This amendment improves the reduction of the temperature from the height of the grid to the height of the site. For the analogous reduction in terms of pressure values, GPT2 reverts to the virtual temperature (i.e., the temperature at which a parcel of dry air would have the same pressure and density as the equivalent parcel of moist air). Information about the humidity of the troposphere is accounted for by the water vapor pressure (see Table 1). The new parameters water vapor pressure, temperature, and lapse rate are beneficial for determining a priori values of zenith wet delays. Furthermore, annual and semi-annual temperature variations can be used for modeling the thermal deformation of VLBI radio telescopes.

**Table 1:** Improvements of GPT2 With Respect to GPT/GMF

	GPT/GMF	GPT2
NWM data	Monthly mean profiles from ERA-40 (23 pressure levels): 1999–2002	Monthly mean profiles from ERA- Interim (37 levels): 2001–2010
Representation	Spherical harmonics up to degree and order 9 at mean sea level	5° grid at mean ETOPO5-based heights
Temporal variability	Mean and annual terms	Mean, annual, and semi-annual terms
Phase	Fixed to January 28	Estimated
Temperature reduction	Constant lapse rate − 6.5°C/km assumed	Mean, annual, and semi-annual terms of temperature lapse rate estimated at every grid point
Pressure reduction	Exponential based on standard atmosphere	Exponential based on virtual temperature at each point
Output parameters	Pressure (*p*), temperature (*T*), mapping function coefficients (*a*_*h*_, *a*_*w*_)	*p*, *T*, lapse rate ( d*T*), water vapor pressure (*e*), *a*_*h*_, *a*_*w*_

This paper describes the development and workings of GPT2 in sections 2 and 3. The new model is compared to GPT and successfully validated on the basis of in situ barometric observations in section 4. Finally, we confirm the improved performance of GPT2 with respect to GPT/GMF within global VLBI solutions (section 4.3).

## 2. Determination of the GPT2 Grid

The proposed empirical model is based on 10 years (2001–2010) of global monthly mean profiles for pressure *p*, temperature *T*, specific humidity *Q*, and geopotential from ERA-Interim [*Dee et al.*, 2011], discretized at 37 pressure levels and 1° of latitude and longitude. After deriving the geometric height information at each vertical level by properly converting geopotential to ellipsoidal heights following Nafisi (2012), we interpolated (or extrapolated) the meteorological parameters *p*, *T*, *Q* to the Earth's surface as needed. The topography is represented by a resampled 1°-version of ETOPO5 (downloaded from http://www.ngdc.noaa.gov/mgg/global/ etopo5.HTML), which comprises orthometric heights at an initial resolution of 5 ′. We assumed linear behavior for computing surface values of temperature and specific humidity, while vertical pressure variations were modeled by an exponential function. For the latter, the exponential coefficient followed either from the height and pressure differences between two levels in case of interpolation (ETOPO5 above the lowest isobaric layer), or from the virtual temperature at pressure level 37 in case of extrapolation (ETOPO5 below the lowest isobaric layer). In addition to the meteorological parameters, we calculated the coefficients *a*_*h*_ and *a*_*w*_ of the hydrostatic and wet mapping functions for every profile using the VMF1-approach as given in Böhm *et al*. (2006b).

The preprocessing yielded 120 monthly values for *p*, *T*, *Q*, *a*_*h*_, *a*_*w*_, and the temperature lapse rate d*T* at each grid point. For all those time series, we estimated mean values *A*_0_ as well as annual (*A*_1_, *B*_1_) and semi-annual (*A*_2_, *B*_2_) variations within a least-squares adjustment, performed separately for each parameter *r*(*t*)


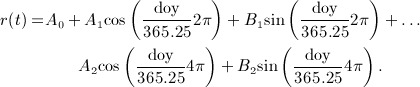
(1)

The estimated values for each grid point were saved to an external ASCII-file (14 MB at 1°, 0.5 MB at 5° resolution), which must be loaded with every run of the GPT2 subroutine. We chose the discretization of 5° and a bilinear interpolation scheme, ensuring sufficiently accurate results (section 3). An internal simulation revealed that these modifications would result in a change in station height estimates exceeding 1 mm for only 1% of all points on a 1° grid, most notably in regions of rapid height variations.

## 3. The GPT2 Subroutine

Using the coordinates of the site and the observation epoch specified as modified Julian date, the GPT2 subroutine calculates local values of {*p*,*T*,d*T*,*a*_*h*_,*a*_*w*_}, as well as an estimate of the water vapor pressure *e* = *Q* ⋅ *p* / 0.622 + 0.378*Q*. Based on the ellipsoidal coordinates (latitude, longitude and height) of the site, the algorithm selects the four nearest grid points around the location and calculates their parameter values by plugging the corresponding means, sine- and cosine-coefficients from the external grid file into equation (1). The model values are valid at the heights of the four grid points. However, pressure and temperature must be corrected for the height difference d*h* between station and grid. For that purpose, at each of the four grid points, the product of d*h* and the temperature lapse rate is added to the temperature value. Analogously to the preprocessing in section 2, the surface pressure is extrapolated exponentially to the station height using the virtual temperature from GPT2. The hydrostatic mapping function coefficient *a*_*h*_ refers to the geoid and the height correction is applied when calculating the mapping function. On the other hand, *a*_*w*_ refers to the mean height of the topography and there is no height correction for the wet mapping function. The final interpolation step from the grid points to the latitude and longitude of the station is realized in a bilinear scheme, which proved to be successful in recovering the spatial variability of each parameter as represented in the original 1° grid.

## 4. Validation of GPT2

In this section, we compare GPT2 to GPT/GMF and specifically test their respective pressure value outputs against barometric observations of 350 sites distributed all over the Earth. The superiority of the new model for usage in geodetic applications is demonstrated by global VLBI solutions.

### 4.1. Numerical Comparison of GPT2 with GPT/GMF

**Figure 1:**
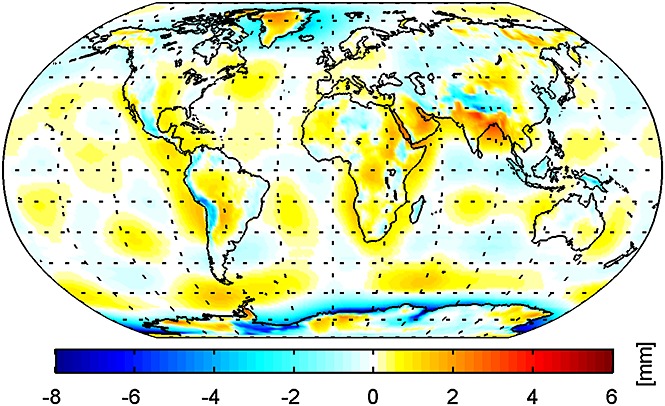
Mean difference of station heights caused by differences in pressure values (GPT2 − GPT) as inferred from the rule of thumb.

In order to compare GPT2 with GPT/GMF, we derived monthly values of {*p*,*a*_*h*_,*a*_*w*_}with a spatial interval of 1°. The gridded differences for each of those parameters can be used to calculate errors in the station height from the slant delay error via the rule of thumb of Böhm *et al*. (2006b), which states that the error in station height is approximately 1/5 of the delay error at 5 ° elevation. This process requires converting the gridded pressure differences to differences in the hydrostatic zenith delay (based on the equation of Saastamoinen (1972)) and mapping them to 5° elevation by means of the wet instead of the hydrostatic mapping function. (The mean difference between hydrostatic and wet mapping function at 5° elevation is 0.6.) From those delay errors due to pressure differences, the bias and standard deviation of the station height errors can be inferred. Figure 1 illustrates the bias at each grid point. The better resolution of GPT2 as well as its refined reduction methods explain the biggest differences of 8 mm in the Antarctic and the considerable effects of about 4 mm over mountain ranges in general. GPT2 certainly brings forth an improved representation of the Antarctic coast, which falls steeply from 2000–3000 m down to sea level. It also exposes artifacts of GPT's spherical harmonic expansion, visible as oscillating patterns at the level of 1 mm over the ocean. Significant differences between GPT and GPT2, in particular in Greenland and the Antarctic, are also due to the change from ERA-40 to ERA-Interim as the underlying source of NWM data [*Dee et al.*, 2011]. Both of these areas are of utmost importance for studies of sea level rise, and providing constraints on estimates of glacial isostatic adjustment by using accurate positions and velocities from GNSS is critical to these studies [*King et al.*, 2010].

**Figure 2:**
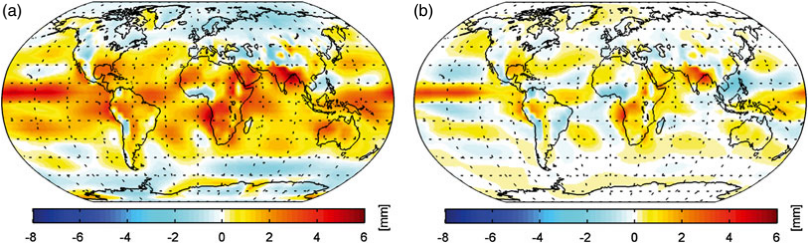
Mean differences (GPT2 − GMF) of station heights caused by different values of (a) the hydrostatic mapping function and (b) the wet mapping function, as inferred from the rule of thumb.

Figure 2a displays the mean differences caused by the change of the hydrostatic mapping function model (GPT2 instead of GMF), expressed as station height errors by applying the rule of thumb. For 12% of the 64800 values the bias is larger than 2 mm and reaches a maximum of 6 mm over the Indian Peninsula. There is a systematic trend from the poles to the equator ( − 1 to +1 mm) caused by the application of a constant Earth radius in ray-tracing for GMF compared to a latitude-dependent Gaussian mean Earth radius for GPT2 [*Nafisi et al.*, 2012]. Figure 2b shows the mean differences due to the different wet mapping function models. Detectable effects up to 5 mm are limited to low latitudes, where dry and rain periods dominate. The spatial distribution of these residuals in the wet mapping function is largely that of the hydrostatic part in Figure 2a near the equator. Possibly, both plots reflect the impact of including semi-annual coefficients and adjusted phases of each parameter in GPT2.

### 4.2. Performance of GPT2 and GPT with Respect to In Situ Pressure Measurements

The International Surface Pressure Database (ISPD [*Compo et al.*, 2011]), which is maintained by the Computational and Information Systems Laboratory (CISL) at the National Center for Atmospheric Research (NCAR), offers access to the history of in situ barometric measurements at land stations and on ships. We downloaded the comprehensive record of station observations for the time span 1990–2010 and selected one arbitrary station at each 9 ° × 9° cell containing observational data. This strategy provided 350 well-distributed stations with barometric measurements over at least 3 years, usually at hourly intervals, but with small sporadic data gaps in the range of a few days. We estimated mean pressure values as well as annual and semi-annual signals within a least-squares adjustment for each station.

**Figure 3:**
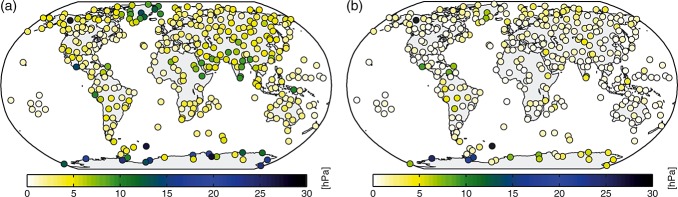
RMS of the differences between in situ pressure observations and pressure values from (a) GPT and (b) GPT2, determined over one climatological year.

The in situ observations and the gridded pressure output from GPT and GPT2 were compared over a period of one climatological year, with the RMS (root-mean-square) of the differences for each station displayed in Figure 3a (GPT) and Figure 3b (GPT2). The new model clearly mitigates the discrepancies existing between GPT and the barometric observations over the entire globe. In particular, GPT2 provides excellent local mean pressure estimates that account for the substantial reduction of RMS values in Greenland, Southern Asia, and at the Antarctic coast. Accordingly, the median of the global RMS field drops from 3.0 hPa with GPT to 1.0 hPa with GPT2. In particular, 0.4 hPa of this reduction is engendered by the inclusion of temperature and water vapor terms, while the main improvement arises from GPT2's enhanced spatial resolution. The newly proposed model offers RMS values below 5 hPa (equivalent to 1.3 mm station height error) for 95% of all stations, whereas in the GPT results, only 80% of the stations are below this threshold. Only five stations in Figure 3b (compared to 10 stations in Figure 3a) yield RMS values above 15 hPa (more than 4.0 mm error in the height). These discrepancies are presumably related to malfunctioning pressure sensors, errors in the quoted in situ heights, or local effects such as inversions, which cannot be resolved with GPT2 either.

### 4.3. VLBI

We ran three global VLBI solutions with the Vienna VLBI Software (Böhm *et al*. 2012) using all suitable 24-hour sessions from 1984.0 to 2012.5. We followed the Conventions 2010 of the International Earth Rotation and Reference Systems Service (Petit and Luzum, 2010) except that we also applied non-tidal atmosphere loading corrections at the observation level (Petrov and Boy, 2004). Whereas the reference solution was determined with the VMF1 and pressure values *p* recorded locally at the sites, two other solutions were calculated with GPT/GMF and GPT2, respectively. There is a clear reduction in the differences with respect to *p* / VMF1 of the mean annual and semi-annual amplitudes of VLBI station heights when using GPT2 compared to GPT/GMF. The mean of the annual amplitude differences decreases from 0.84 to 0.55 mm, and the mean of the semi-annual amplitude differences decreases from 0.75 to 0.47 mm when using GPT2 instead of GPT/GMF.

**Figure 4:**
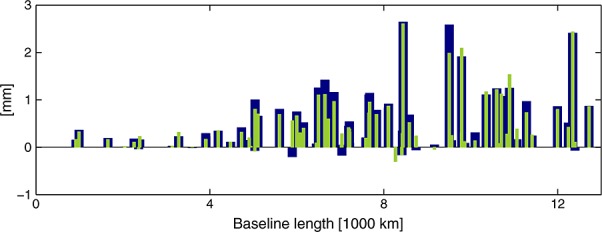
Improved baseline length repeatabilities with instantaneous local pressure values and the VMF1 compared to GPT/GMF (blue) and GPT2 (green).

In terms of baseline length repeatabilities, the solution deploying *p* / VMF1 is clearly superior to the empirical models. However, as shown in Figure 4, for 60% of the baselines (42 out of 70 baselines which are observed in more than 200 sessions), the repeatabilities are better with GPT2 compared to GPT/GMF.

## 5. Conclusions

In this paper, a new empirical slant delay model called GPT2 has been presented. This model is an improved version of the existing models GPT/GMF with reduced horizontal resolution (5°), enhanced temporal variability, and additional parameters, i.e., vertical temperature gradient and water vapor pressure at all grid points. The latter parameters are useful when a priori values of zenith wet delays are required. The benefits and the improved performance of GPT2 with respect to the previously recommended models GPT/GMF have been illustrated by comparing the models directly, by validating them against in situ barometric observations, and by analyzing station height estimates from VLBI. We recommend the replacement of the old GPT/GMF with GPT2 as empirical model in the analysis of radio space geodetic observations. In particular, results with GPT2 will be more meaningful for geophysical studies such as hydrological investigations for which annual and semi-annual height variations are of interest.

However, there are some technical changes, which must be considered for the transition from the old GPT/GMF: the mapping function coefficients are provided by GPT2 and can be used directly as input to the subroutine for the gridded Vienna Mapping Function, i.e., to *vmf*1_*ht.f*. Hence, there is no “GMF2” but just GPT2. Moreover, the new subroutine is called only once per 24-hour session since it contains only annual and semi-annual variations of the parameters. Thus, the adopted concept is to allow just one epoch in the input arguments, as values from multiple stations can be specified with one call.

## Supplementary material

All Supporting Information may be found in the online version of this article.
